# Prevalence of bluetongue virus antibodies and associated risk factors among cattle in East Darfur State, Western Sudan

**DOI:** 10.1186/2046-0481-67-4

**Published:** 2014-02-07

**Authors:** Hadia OM Khair, Ibrahim A Adam, Shakir B Bushara, Kamal H Eltom, Nasreen O Musa, Imadeldin E Aradaib

**Affiliations:** 1 Molecular Biology Laboratory (MBL), Department of Clinical Medicine, Faculty of Veterinary Medicine, University of Khartoum, P.O. Box 32, Khartoum North, Sudan

**Keywords:** Epidemiology, Survey, Orbiviruses, BTV, cELISA, Sudan

## Abstract

**Background:**

Bluetongue virus (BTV) is an insect-transmitted virus, which causes bluetongue disease (BT) in sheep and a fatal hemorrhagic infection in North American white-tailed deer. However, in cattle the disease is typically asymptomatic and no overt clinical signs of disease appear to be associated with BTV infection. Serological evidence and isolation of different BTV serotypes have been reported in Sudan, however, no information is currently available in regard to previous exposure of Sudanese livestock to BTV infection in East Darfur State, Sudan.

**Aims:**

To determine the prevalence of BTV antibodies and to identify the potential risk factors associated with BTV infection among cattle in East Darfur State, Sudan.

**Methods:**

A total of 224 blood samples were collected randomly from five localities in East Darfur State, Sudan. The serum samples were screened for detection of BTV-specific immunoglobulin G (IgG) antibodies using a competitive enzyme-linked immunosorbent assay (c-ELISA).

**Results:**

Serological evidence of BTV infection was observed in 150 out of 224 animals accounting for a 67% prevalence rate among cattle in East Darfur State. Older cattle (>2 years of age) were six times more likely to be infected with BTV (OR = 6.62, CI = 2.87-15.26, p-value = 0.01). Regarding animal source (contact with other herds) as a risk factor, it was shown that cattle purchased from market or introduced from other herds were 3 times at higher risk of being infected with BTV (OR = 3.87, CI = 1.07-13.87, p value = 0.03). Exposure of cattle to the insect vector increased the risk of contracting BTV infection by six times compared to non-exposed cattle (OR = 6.44, CI = 1.53-27.08, p value = 0.01).

**Conclusion:**

The present study indicated that age, animal source and the intensity of the insect vector are influential risk factors for BTV infection in cattle in the Darfur region. Surveillance for BTV infection should be extended to include other susceptible ruminants and to study the distribution of the insect vectors to better predict and respond to a possible BTV outbreak in the State of East Darfur, Sudan.

## Background

Bluetongue (BT) virus (BTV), the cause of BTV infection, is an insect-transmitted virus, which belongs to the *Orbivirus* Genus in the family *Reoviridae *[1-3]. The virus has a worldwide distribution and exists in at least 26 distinct BTV serotypes including serotypes 25 and 26, which were identified recently [4,5]. In Sudan, previous studies have shown that the seasonal incidence of BTV is a predictable event related to the rainy season [6]. BTV is transmitted by different species of *Culicoides* midges. In the Sudan, *Culicoides imicola* was reported to be the principal insect vector for transmission of the virus [6-9]. At least, five BTV serotypes designated as serotypes 1, 2, 4, 5 and 16 are enzootic in different States of the Sudan. These serotypes were recovered from sentinel calf herds at the Khartoum University farm, Shambat; Nyala, South Darfur State, as well as from Umbenin, Sennar State, Sudan [10,11].

Bluetongue virus may cause a severe hemorrhagic disease in certain breeds of sheep and an often fatal hemorrhagic infection in North American white-tailed deer. Other sheep breeds, such as Sudanese ecotypes of sheep, may develop only febrile disease while cattle and camelids mostly develop subclinical infections [12]. The indirect losses associated with decreased body weight and condition, drop in milk production, and poor subsequent reproductive performance were thought to have a greater economic effect than occasional overt disease [8,10,13]. In addition, there is restriction on the international trade of livestock and associated germplasm from BTV-endemic countries, unless the animals are certified free of infection by conventional virus isolation or serology [13-15]. Such a restriction could lead to economic losses for BTV-endemic countries, like Sudan, which rely on the sale of livestock for foreign exchange.

Currently, little is known about the prevalence of BTV infection in Darfur, and no information is available in regard to the potential risk factors associated with the infection in Sudan. Previous studies on experimental BTV inoculation or evaluation of BTV infection in sentinel cattle herds showed that infected cattle developed viremia and seroconverted. Thus, cattle can act as reservoir for the virus enabling transmission to highly susceptible sheep. This observation suggested that cattle may play an important role in the epidemiology of BTV [6,16-18]. The presence of BTV-specific antibodies and subsequent recovery of several BTV serotypes from cattle have been reported in various regions of Sudan [7,8,10,18,19]. It is, therefore, becoming increasingly obvious that the control of this arboviral infection is important in the Sudan given the large numbers of livestock in the country, and their importance to the national economy and rural communities [20]. Epidemiologic studies including implementation of improved surveillance are urgently needed to better predict and respond to this important viral pathogen in the Sudan [6,11]. The objectives of the present study were to estimate the prevalence of BTV-specific IgG antibodies and to identify the potential risk factors associated with BTV infection among cattle in East Darfur State, Sudan.

## Methods

### Study area

**Figure 1:**
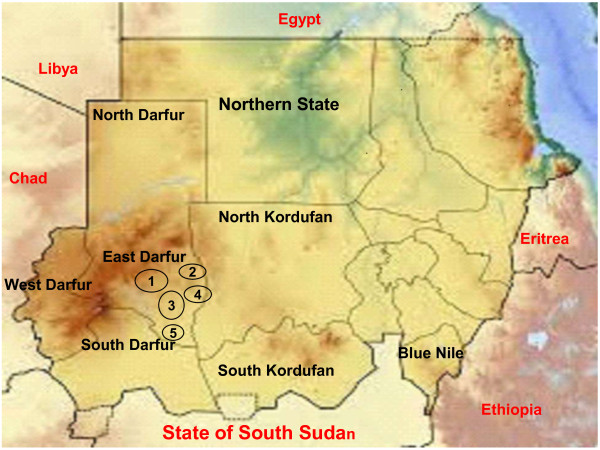
**Localities of East Darfur are illustrated by circle with the numbers inside.** Number 1 = ElDeain; Number 2 = Abujabra; Number 3 = Assalaysa, Number 4 = Elfirdus, Number 5 = Bahr El Arab.

The State of East Darfur is located between latitudes 12–9.5 N - longitude 25 to 28 E and altitude of 449 meters (1476 feet) above sea level and about 831 km from the capital of Sudan, Khartoum. The State is boarded by North Darfur State in the north; Northern Kordufan in the east, South Darfur in the west and the State of South Sudan to the South. The total population in East Darfur is approximately 0.3 million as estimated in 2006. The livestock population in East Darfur constitutes one of the major sources of the income to rural communities and the national economy, at large. This State is one of the main animal rearing areas in the country and possesses the large numbers of livestock. The cattle population of this state is presently estimated to be as 2–3 million. About 80% of cattle in this State are kept under nomadic system, where nomads migrate to the State of South Sudan in the dry season and back to north in the rainy season. During this movement the nomads cover the area from borders of the State of South Sudan up to North Darfur, where the animals inter mix freely and share common water, pasture route and premises with cattle in the State of South Sudan. In Figure 1, a map of the localities included in the study area of East Darfur State is presented.

### Study design

A cross sectional study was conducted to estimate the prevalence rate of BTV-specific IgG antibodies in cattle and to investigate the potential risk factors associated with the disease. The multistage probability sampling method was conducted. Five localities were randomly selected from all nine localities in the State of East Darfur. Two administration units were selected from each locality. Seven villages were selected from each unit. Finally, simple random sampling was applied to choose the animals from each herd [21]. The study is reported in compliance with the STROBE statement [22].

### Questionnaire

A pre-tested structured questionnaire with the primary objective of elucidating the multifactorial background of disease was conducted in an interactive manner at all selected herds. All animals included in this study were subjected to a questionnaire, which was filled out by the animal owners. The questionnaire included individual risk factor attributes including age (younger animals < 2 years, older animals 2 years and above), sex (male, female), breed (indigenous, cross), body condition (thin, emaciate, fat), and management risk factor attributes including herd size (small, medium, large), grazing system (nomadic, semi-nomadic, stationary), insect vector (presence or absence), the source of each animal in the herd (raised on farm, purchased from other farms or purchased from local market) and the five localities included in the study (Additional file 1).

### Ethical clearance

The blood collection procedure from cattle was performed by qualified veterinarians following proper physical restraint of animals to ensure both personnel and animal safety. Livestock owners were explained the study purposes and procedures and upon agreeing to participate, they provided a written consent prior to study procedures and blood collection from their animals. The study received ethical clearance from the Research Board of the College of Veterinary Medicine, Sudan University for Science and Technology, Khartoum, Sudan. The risk factor information was obtained from the animal owners through the questionnaire form, which permitted use of the blood samples for diagnostic and research purposes.

### Collection of blood samples

A total of 224 blood samples were collected from cattle in East Darfur State, Sudan. Serum samples were separated from cattle blood and were kept frozen at -20°C until used for detection of BTV-specific IgG antibodies using competitive enzyme-linked immunosorbent assay (cELISA).

### Competitive Enzyme-Linked Immunosorbent Assay (cELISA)

A competitive enzyme-linked immunosorbent assay (cELISA) was performed using a commercially available BTV antibody cELISA Kit (Veterinary Medical Research and Development Laboratory, USDA Product Code 5010.20 (Pullman, WA, USA). The sera were screened for BTV-specific IgG antibodies basically as described by the manufacturer’s specifications. cELISA was performed in 96-well antigen-coated microplates. Unless stated otherwise, 25 or 50 microliters (μl) test volumes were used in the cELISA assay. The incubations were performed for 15 min at room temperature (23 ± 2°C). The plates were washed three times with the provided washing buffer. Briefly, aliquots of 25 μl test sera as well as positive and negative controls sera were transferred undiluted to the BTV antigen coated plates, using multi-channel pipette. After incubation, the plates were washed, and 25 μl of antibody-peroxidase conjugate were added to each well. The plate was then incubated at 15 min at room temperature. The plates were then washed and 50 μl the substrate was added to each well. The reaction was stopped using 50 μl of the stopping solution. The results were read either visually or by using ELISA reader set at 630 nm. A presumptive diagnosis was made when the test samples produced an optical density < 50% of the mean of the negative controls. The test samples were considered negative if the optical density ≥ 50% of the mean of the negative controls.

### Statistical analyses

The data were entered in computer using statistical package for social studies (SPSS) software package for Windows (version 16.0) and double checked before analyses. The data collected were entered into a computer on a Microsoft Excel spreadsheet. Statistical analysis was performed using ‘Statistical package for the social sciences’ (SPSS), version 16 (for Windows). Associations between the outcome variable (status of BTV infection in the cattle) and its potential risk factors were first screened in a univariable analysis using Chi-square test. Potential risk factors with *P* value *<* 0.25 (two tailed; *α* = 0.25) were initially considered significant in *χ*^2^ test. The significant results of the univariable analysis were reentered in the final model using multivariable analysis. A multivariable model for the outcome variable was constructed using manual stepwise forward logistic regression analysis. BTV infection was considered as the dependent variable and the risk factors as independent variables. Finally, odd ratios and 95% confidence interval (CI) were calculated, and risk factors with a *p-*value < 0.05 were taken as significant association to BTV infection.

## Results

Using a cELISA, BTV-specific IgG antibodies were detected in 150 (67%) of 224 cattle included in the study. The overall prevalence rate of BTV antibodies among cattle in the East Darfur State of Sudan was estimated to be 67%. The highest and lowest rates of BTV seropositivity were recorded in Bahr Elarab and Asalaya localities, respectively. Initially, univariate analysis using Chi-square test was conducted for the association between the potential risk factors and BTV infection. In Table 1, the results of the univariate analysis showed that the independent variables including, age, sex, breed, animal source, presence of insects and locality were statistically significant. The risk factors that were significant in the univariable model were re-entered into final multivariate model using logistic regression analysis. In the final models, a variable with a P-value <0.05 was considered statistically significant. In Table 2, the final models of BTV infection indicated that only three independent risk factors were statistically significant. There was no significant association between rates of BTV infection among cattle and the potential risk factors including animal sex, breed, grazing system, herd size, body condition and localities. In contrast, when potential risk factors were measured against BTV infection rates, older cattle (>2 years of age) were six times more likely to be infected with BTV (OR = 6.62, CI = 2.87-15.26, p-value = 0.001). Cattle purchased from market or introduced from other herds were 3 times at higher risk to be infected with BTV (OR = 3.87, CI = 1.07-13.87, p value = 0.03). Exposure of cattle to insect vector increased the risk of contracting BTV by six times compared to non-exposed cattle (OR = 6.44, CI = 1.53-27.08, p value = 0.01).

**Table 1:** Univariate analysis for the association between potential risk factors and BTV infection among cattle in East Darfur State of Sudan using Chi-square test

**Risk factors**	**Animals tested**	**Animals affected (%)**	**df**	***χ*** **2**	**p-value**
Locality			4	18.77	0.01
Aldeain	60	31(51.6%)			
Abugabra	44	25(56.8%)			
Bahrelarab	94	70(74.4%)			
Asalaya	10	10(100.0%)			
Fardous	16	14(87.5%)			
Age			1	18.69	0.01
Small	72	34 (47.2%)			
Old	152	116(76.3%)			
Sex			1	9.25	0.02
Female	170	123(72.3%)			
Male	54	27(50%)			
Breed			1	9.07	0.03
Endogenous	207	133(64.2%)			
Cross	17	17(100.0%)			
Body condition			1	0.30	0.58
Thin	206	139(67.5%)			
Fat	18	11(61.1%)			
Animal source			2	17.17	0.01
Raised on farm	166	110(66.3%)			
Purchased from other farms	36	32(88.9%)			
Purchased from local market	22	8(36.4%)			
Grazing system			1	1.29	0.25
Stationary	94	59(62.7%)			
Nomadic	130	91(70%)			
Herd size			2	5.14	0.08
Small	40	26(65%)			
Medium	83	63(75.9%)			
Large	101	61(60.4%)			
Insect presence			1	7.48	0.01
No	48	17(35.4%)			
Yes	176	133(75.5%)			

**Table 2:** Multivariate analysis, using logistic regression model, for significant association (p > 0.05) of risk factors and BTV infection among cattle in East Darfur State, Sudan

**Risk factors**	**OR**	**95.0% C.I**	**p-value**
Age	6.62	2.87-15.26	0.01
Insect vector	6.44	1.53-27.08	0.01
Contact with other animals	3.87	1.07-13.87	0.03

## Discussion

In recent years, the global distribution and nature of BTV infection has changed significantly. Climate change has been implicated as a potential cause of this dramatic event observed globally. BTV has become of great veterinary concern to dairy producers, wildlife managers and veterinary diagnosticians because of the frequent occurrence of outbreaks among domestic and wild ruminants in geographical regions previously known to be BTV-free [23-25]. However, in areas of endemicity, as in the case of the Sudan, overt clinical disease has never been reported among naturally infected cattle [6,8,11,26-29]. Bluetongue infection constitutes one of the major unresolved veterinary problems in certain breeds of sheep and in North American white-tailed deer [12,30]. Very little information is available about the epidemiology of BTV in the Middle East and East Central Africa including the Sudan. To advance beyond the current knowledge of the epidemiology of the disease, we conducted this study to determine the prevalence of BTV infection and associated risk factors among cattle in East Darfur, the largest producing live stock region in the country. A lot of research efforts have been made to facilitate rapid molecular detection and differentiation of BTV and BTV-related viruses in susceptible ruminants [11,26,29,31-36].

Previous epidemiological surveys showed high prevalence rates for BTV seropositivity in Iran (93.5%) and Southern Turkey (88%) [37,38]. In Sudan, earlier serological surveys indicated that BT infection is generally widespread and occurs in all domestic ruminants, with as high as 61.5% prevalence rate in Juba, South Sudan [18]. Subsequent serological survey for antibodies against BTV infection in cattle in Khartoum State showed high prevalence rate of 51.1% among the examined animals [19]. In the present study, the seroprevalence of BTV group-specific antibodies in cattle of East Darfur (67%) is markedly higher than previously reported prevalence rates in other states of Sudan. The high seroprevalence rate of BTV in East Darfur State (67%) could be attributed to the construction of irrigation projects and petroleum industry in the region.

The petroleum industry is associated with pumping of large quantity of water onto the surrounding environment. The irrigation projects and petroleum-associated water might contribute in providing suitable habitats and humidity sources for breeding of the insect vector. These environmental changes provide suitable climatic condition for survival of the adults and larvae of *Culicoides* vectors in this region [6]. In contrast, the locality of Dongla, in the Northern State of Sudan, was reported to be BTV-free zone. This is mainly due to the hot and dry climatic conditions in the region, which render the environmental conditions unfavorable for the activity and maintenance of the life cycle of the insect vector [18].

In the present study, the prevalence of BTV infection (67%) is comparable to those reported amongst cattle in Juba, South Sudan where substantial rainfall events and high density of the insect vector are well documented [18]. The presence of BTV infection in Sudan and the risks these infected cattle pose for native sheep, necessitates the importance of improved surveillance system for this viral pathogen in Sudan. The BTV infection rates increased with the increasing of age in the studied herds. When assessing age as a risk factor, there was a significant association between the BTV infection rate and the age of the animal. It was shown that the calves started to get infected with BTV after the age of 2 years. At this age, the animals are usually released into the pasture for grazing, where they are likely to be exposed to infected vectors and subsequent BTV infection. We believe that the association of BTV infection and age is probably attributed to frequent exposure of older cattle to infected *Culicoides* vectors. Young calves (<2 years) are usually kept indoors and are well taken care of by the animal owners from contracting infectious diseases, particularly the insect and tick-borne infections [20]. Our result is in agreement with previous epidemiological surveys, which reported higher risks of older animals for BTV infections [39]. It should be noted that the BTV-specific antibodies detected among cattle in East Darfur State indicate natural infection as there is no vaccination program for the disease in the country. In addition, all cattle included in this study were aged over one year. Therefore, it is assumed that maternal antibodies were no longer persisted and that antibody indicated natural infection with BTV. In addition, animal source (contact with other herds) is another factor that affects BTV seropositivity results. It is probable that introducing infected animals into the herd would allow the local midge population to become infected, with a subsequent increase in BTV infection rates. Our result is in agreement with that of the study conducted by Mohammadi who reported a significant association between BTV infection and contact with other herds among animals in Fars province, Iran [39]. Regarding the management risk factors, there was a significant association between BTV infection and the exposure of cattle to the insect vector.

In contrast, the risk assessment studies indicated that there was no significant association between BTV infection and the rest of the individual or management risk factors included in the study. It is worth mentioning that the animal sex has no association with BTV infection among male and females as both sexes are equally infected with BTV. In the present study, there was no significant difference between localities and BTV infected cattle. However, wind-borne insects are believed to have also been responsible for the recent spread of BTV from North Africa to southern Europe, and between Mediterranean islands, in some instances involving distances of up to several hundred kilometers. Infected *Culicoides* vectors can be carried by the wind over a long distance to susceptible livestock [40,41]. Recently, it has been reported that the effects of global climate change have facilitated the spread of epizootic hemorrhagic disease virus (EHDV), a closely related orbivirus, into regions of the world in which the virus has never been reported before [23]. Highest and lowest rates of BTV infections were recorded in Bahr Elarab and Asalaya localities, respectively. The high level of BTV infection in Bahr Elarab is attributed to the substantial rainfall events and the high density of the insect vector in that particular locality.

## Conclusions

Bluetongue virus does exist in East Darfur State, Sudan, as determined by the presence of BTV-specific IgG antibodies. Nevertheless, an outbreak of BTV among native sheep in Darfur is yet to be reported. The specific BTV serotypes circulating in the region remain unidentified. Future surveillance systems for BTV infection should be extended to include other susceptible animals such as sheep, goats and camels. In addition, the distribution of *Culicoides* vectors in the region should also be considered to better predict and respond to BT infection in the State of East Darfur, Sudan.

## Competing interests

The authors declare that they have no competing interests.

The result of this study does not reflect the opinion of the funding sources.

All authors have read and approved the final version of this manuscript.

## Authors’ contributions

HOMK optimized the ELISA for detection of BTV-specific IgG antibodies in cattle sera, design the experiment, and prepared the draft manuscript. IAA and SBB collected the blood and prepared the serum samples and helped with SPSS analysis. KHA and NOM designed the study. IEA designed the experiment and prepared the final manuscript. All authors read and approved the final version of the manuscript.

## Supplementary Material

Additional file 1 **Questionnaire.** Investigation of Bluetongue disease among cattle in East Darfur State. Click here for file
