# Supplementary material for: Prevalence of bluetongue virus antibodies and associated risk factors among cattle in East Darfur State, Western Sudan
Source: Ir Vet J. 2014 Feb 7;67(1):4. doi: 10.1186/2046-0481-67-4 (PMC4016605; doi:10.1186/2046-0481-67-4)
Supplement: Additional file 1 — Questionnaire. Investigation of Bluetongue disease among cattle in East Darfur State. [file 2046-0481-67-4-S1.doc]

**Questionnaire**

**Investigation of Bluetongue disease among cattle in East Darfur State**

**Locality__________________ date _________________ Herd Owner___________________ Herd Code ____________________ Address ______________________________________**

**I. Individual risk factors**

**1-Age (years)**

<2 (    )

2-4 (    )

>4 (    )

**2-Sex**

Male (    )

Female (    )

**3-Breed**

Endogens (    )

Cross (    )

**4-Body condition**

Emaciation (    )

Thin  (    )

Fat (    )

5- Source of animal:

Raised on farm (    )

Purchased from other farms (    )

Purchased from local market (    )

**II. Management Risk Factors**

**6-Grazing system**

Nomadic (    )

Semi nomadic (    )

Stationary (    )

**7-Herd size**

Small  (    )

Medium (    )

Large (    )

**8- Insect**

Yes (    )

No (    )

**9- Locality**

1. Aldeain (    ) 2. Abugabra (    )

3. Bahrelarab (    ) 4. Asalaya (    )

5. Fardous (    )
